# Evaluating the support and associated strain distribution in unilateral obturator with different designs: An experimental and finite element study

**DOI:** 10.1371/journal.pone.0321710

**Published:** 2025-05-09

**Authors:** Mohammed Mousa, Adam Husein, Mohamed El-Anwar, Norwahida Yusoff, Hussein Alhelay, Badi Alazhari, Fadhel Alsharari, Bader Alzarea, Mohammed Sghaireen, Johari Yap Abdullah

**Affiliations:** 1 Prosthetic Dental Sciences, College of Dentistry, Jouf University, Sakaka, Saudia Arabia; 2 School of Dental Sciences, Universiti Sains Malaysia, Kubang Kerian, Kelantan, Malaysia; 3 Department of Preventive and Restorative Dentistry, University of Sharjah, College of Dental Medicine, Sharjah, UAE; 4 Department of Mechanical Engineering, National Research Centre, Giza, Cairo, Egypt; 5 School of Mechanical Engineering, Universiti Sains Malaysia, Penang, Malaysia; 6 Mahayel Specialist Dental Center, Aseer Health Cluster, Asser, Saudi Arabia; 7 Dental Research Unit, Center for Transdisciplinary Research (CFTR), Saveetha Dental College, Saveetha Institute of Medical and Technical Sciences, Saveetha University, Chennai, India; University of Puthisastra, CAMBODIA

## Abstract

This study aimed to evaluate support (the resistance to tissue-ward movement) and strain distribution in unilateral obturators with four designs using Digital Image Correlation (DIC) and Finite Element Analysis (FEA). Twelve epoxy-resin models were prepared to receive removable obturators that have four designs, including acrylic resin-based obturators (ARO), linear (LDO), tripodal (TDO), and a newly modified one termed fully tripodal design obturator (FTDO) were used for DIC. The models were installed in a DIC set to receive a vertical load of 150N. The strain on the dentate and defect sides was evaluated using DIC software. Mathematically, four finite element models were prepared to receive vertical and lateral loads of 100N on two points. The support and strain were assessed using the ANSYS workbench. Using DIC, the ARO demonstrated the highest strain values on the defect area and as an entire prosthesis, followed by the LDO on the defect side. Using FEA, the TDO produced the highest strain value with anterior (oblique) and posterior loads. LDO produced the lowest support and highest strain on the anterior teeth compared to TDO and FTDO. ARO resulted in the highest total strain, while the TDO produced the lowest. Both TDO and FTDO were comparable in terms of strains and support.

## Introduction

Obturators serve as a treatment option for patients who have had a maxillectomy, mainly when implant-supported prostheses are not viable [1–4]. Among the six classifications proposed by Aramany, class I pertains to unilateral defects that reach the middle of the palate without involving the entire premaxilla [5]. To address this defect, practitioners commonly use linear design (LDO) and tripodal design (TDO) beside the acrylic resin-based (ARO) obturators [6,7]. The key difference between these two types lies in their source of support: the LDO relies on the posterior teeth for support, while the TDO gains support from both the anterior and posterior teeth [6,7].

Biomechanics has potential clinical and biological importance in removable obturators, as it allows for the characterization of supporting structures and prosthetic restoration. One of the essential characteristics of biomechanics is stress and strain. Stress is the applied force on a material, while strain is the deformation of a material because of an applied force [8,9]. Various bioengineering tools have been utilized to assess the biomechanical behavior of supporting structures under prostheses, such as strain gauges, deflectometers, linear differential transformers, digital image correlation (DIC), and finite element analysis (FEA). DIC is a full-field strain measuring technique using the optical-numerical approach to determine the displacement, deformation, and surface tension in nonhomogeneous and anisotropic materials [10–12]. The DIC has limitations, including being less precise than the other techniques, the need for meticulous surface preparation and careful optimization of the specimens, the limited depth of the strain evaluation, and adequate optical access to the specimen [12]. FEA offers low cost, specimen standardization, simulation of complicated scenarios, and the capacity to identify potential failure areas [13]. The limitations of the FEA include the lack of consideration of the clinical factors, the accuracy of the models, the precise input data, and the experience of the researchers [14]. For many reasons, including the sensitivity to the oral environment and the difficulty of implementing complex defects such as maxillofacial defects, and considering the biological influences of the oral cavity, no single method could fulfill the complete requirements to display the biomechanical behavior thoroughly [12,15–19].

The strains were evaluated in the various forms of removable prosthodontics, including complete and partial dentures. The removable complete dentures have reported fractures at the midlines due to the generation of the strain at the midline of the prosthesis. That strain is folded in the palateless dentures [20]. Adding implants decreased the strain within the denture base and underlying structure. With more implants added, less strain will be generated on the midline and anterior implants [21,22]. In addition, adding metal reinforcement to the denture base decreased the strain production in the middle of the denture base [23]. The removable partial denture major and minor connectors were found to be subjected to stress and deformation [24]. The type, length, and thickness of connectors primarily influence the deformation in removable partial dentures. A more rigid major connector results in less base deformation than a smaller thickness or weaker materials [24]. As the saddle length increases, the displacement and the deformation will increase, especially in the posterior portion of the saddle [24].

Due to the shape and extension of the maxillary defect, the supporting structures and obturators are subjected to massive deformation in the forms of stress and strain [25]. That may result in losing the supporting bone and abutments with final prosthesis failure [26]. Although DIC was used in prosthetic dentistry two decades ago, its uses were mainly focused on implant-assisted prosthetics [11,19,27,28]. FEA, instead, has been used to evaluate stress and displacement in various prostheses in the last two decades [29–33].

As the authors are aware, there is a lack of literature regarding evaluating the support (tissue ward movement) and associated strain of the obturators used to treat Aramany class I. Also, designs showing mechanical and biological benefits in support and strain that may add new options to prosthodontics were needed. That was the aim of the current study. The null hypothesis stated no differences in the tissue-ward movement (support) and strain in the obturators with the assigned designs.

## Materials and methods

Following approval from the “Ethics and Research Committee, USM” under reference number USM/JEPeM/21030222, the study was carried out at the Schools of Dental Sciences and Mechanical Engineering at Universiti Sains Malaysia. Using DIC and FEA, the study assessed the support and strain distribution of obturators featuring four designs: acrylic resin-based obturator (ARO), linear design (LDO), tripodal design (TDO), and fully tripodal design obturators (FTDO).

**Fig 1:**
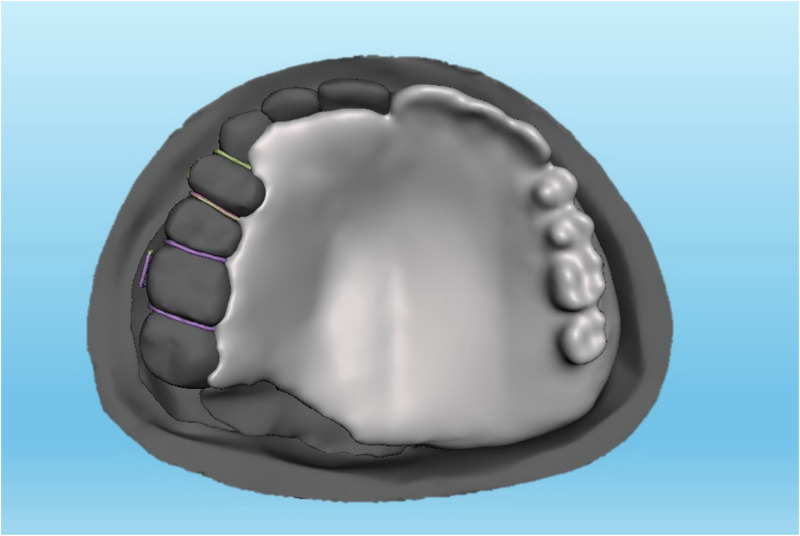
A Meshmixer model of acrylic resin-based obturator.

**Fig 2:**
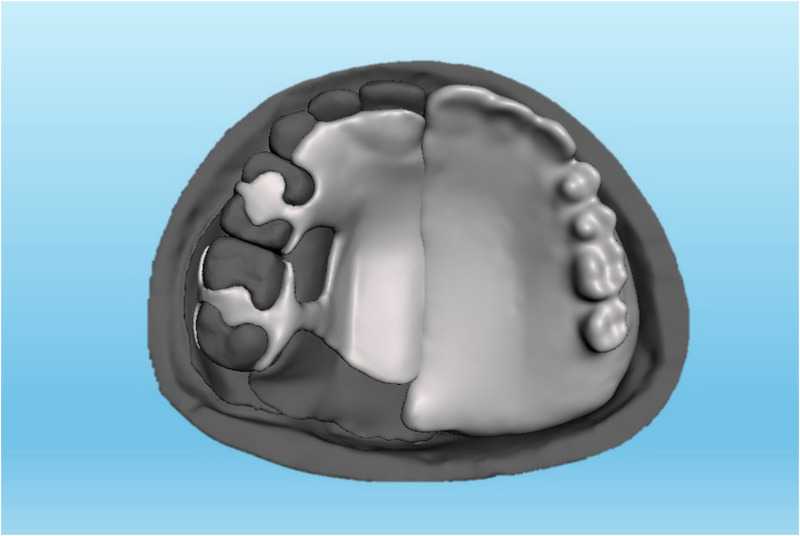
A Meshmixer model of linear design metal-based obturator.

**Fig 3:**
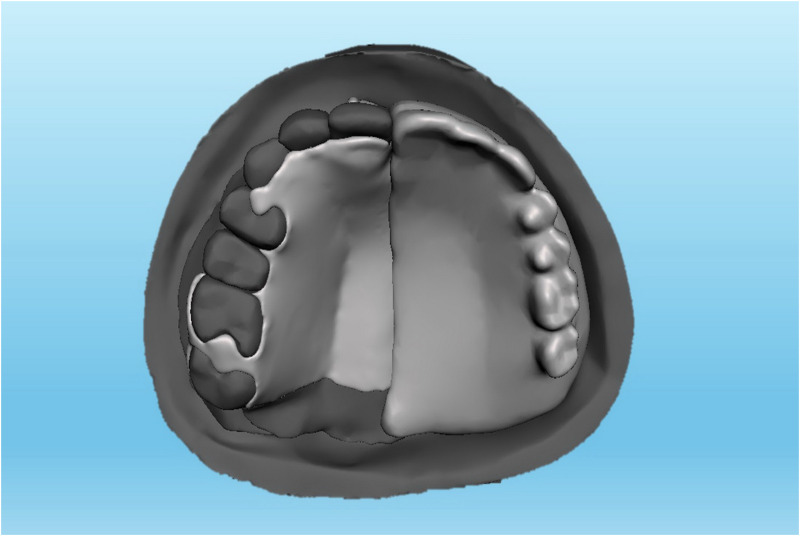
A Meshmixer model of tripodal design metal-based obturator.

**Fig 4:**
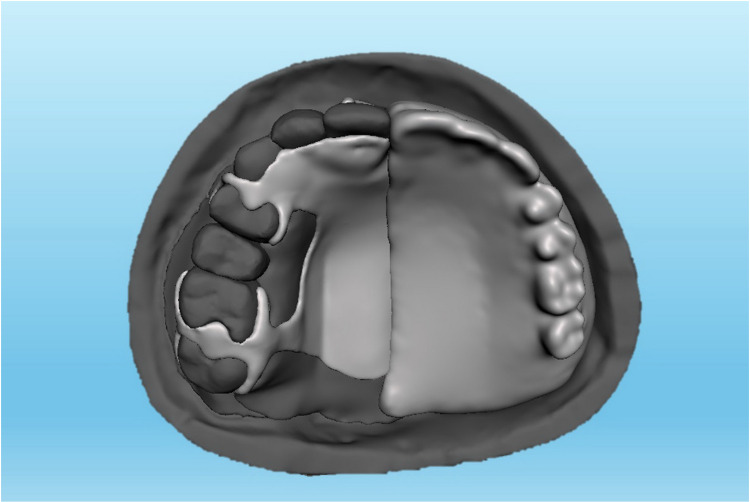
A Meshmixer model of the fully tripodal design metal-based obturator.

The data of the current study was collected from a computerized tomography scan of a 37-year-old Malaysian male who had undergone unspecified brain surgery. The data was imported into Mimics software (Mimics 17.0: Materialize; New York, USA) to create a model of unilateral maxillary defect on the left side while the right side was kept intact. The developed model was refined using Meshmixer software (Meshmixer 5.3.4: Autodesk Inc.; California, USA) and then printed using a 3-dimensional printer (Ender-3 S1: Shenzhen Creality 3D Technology Co.; Shenzhen, China). A 3 mm thick, soft ethyl-vinyl-acetate sheet (Erkoflex Soft splint 3 × 120: Erkodent Erich Kopp; Pfalzgrafenweiler, Germany) was softened and applied to the palate and defect area of the model to demonstrate the mucosa [34]. Following boxing the model using baseplate wax (Cavex modeling wax; Cavex), twelve impressions were made using silicone duplicating materials (Replisil 22S: Silconic; Baden-Württemberg, Germany). The teeth of the right side were segmented, printed, and replicated into 12 sets of hard acrylic copies (Extra-hard self-cure; Vertex dental; Soesterberg, Netherlands) using an index of rubber materials (Flexceed Kit: GC Flexceed; Dublin, Ireland). The periodontal ligament was simulated by applying 0.1-0.3 mm of polyvinyl siloxane impression material (Flexceed Kit (putty type): GC Flexceed; Dublin, Ireland) on the root of the teeth to cementoenamel junction [35]. A 3 mm thick, soft ethyl-vinyl-acetate sheet (Erkoflex Soft splint 3x120; Erkodent Erich Kopp; Pfalzgrafenweiler, Germany) was shaped and adapted to the twelve duplicated impressions to simulate the oral mucosa, and then the acrylic teeth were then repositioned to their respective positions. The models were poured with clear epoxy resin (Clear epoxy: Craft E.; Kelantan, Malaysia) and left for 24 hours for complete hardening. Indexes with rubber materials have been made around the crown of the teeth before extracting them from their models to provide guidance when relocating the teeth during periodontal simulation. The teeth on the dentulous side were then split using a thin metal disc, the models were submerged under hot running water for about 5 minutes, and the teeth were extracted using suitable forceps. Thereafter, the rubber materials adapted around the roots were wiped off, and the sockets were cleared of rubber remnants. A mix of soft clear epoxy resin (Clear soft epoxy; Craft E. Kelantan, Malaysia) was prepared and poured into the cleared sockets, and the teeth were then replaced at their respective sites using the previously prepared indexes. Twenty-four hours later, the abutments were ready to receive the assigned prosthesis, including ARO, LDO, TDO, and FTDO. Twelve obturators were fabricated, including 3 for every design [19,36]. The number of models was chosen due to the three sides (dentate, anterior, and edentulous sides) for which the videos and sequential photos were taken. For ARO, the retention was provided by two Adams clasps on the first premolar and molar (Fig 1) [37]. For the LDO, the support was provided by two occlusal rests on the distal sides of the first premolar and molar and two occlusal rest seats on the mesial sides of the second premolar and molar (Fig 2) [33]. The support of the TDO and FTDO was the same, obtained by two cingulum rests on the central incisor and canine and occlusal rests on the first premolar, first, and second molar, as stated in the literature [6,7]. For retention, the TDO had two clasps on the anterior and molars, while the FTDO had three on the anterior, first premolar, and molars (Figs 3, 4). The major connectors in the TDO covered the entire palate, while those of FTDO covered less palatal tissue. After finalizing the frameworks and checking them on their corresponding models, the prosthetic portion was made using self-cure acrylic resin (Vertex: Vertex dental; Soesterberg, Netherlands).

### Evaluation of strain using DIC

To evaluate the strain using DIC, the outer surface of the models was roughened using sandpaper. The models with the respective prostheses were installed in the DIC setup, which is composed of a universal testing machine (Instron 3367; Instron; Norwood, USA) controlled by software (BlueHill2: Instron; Norwood, USA), a CCD camera with a resolution of 1626 × 1236 pixels (CCD Imager Pro-X; Lavision; Ypsilanti, USA), a white light source, a desk computer, and DIC software (DAVIS 8.1.1: Lavision; Ypsilanti, USA). The testing machine was adjusted to be less than 1 mm from the model, the camera was turned to live mode, and its resolution was manually adjusted until the model became apparent on the screen. Depending on previous studies, a load of 150N was placed on the region of the central and molar areas of the obturators [30,33]. Sequential photos and videos of the dentate and defective side were captured during load application. Three records were made for each model to evaluate the elaborated strain in the supporting tissue of the anterior teeth, the dentate, and defective sides. The records included one to the anterior teeth during the anterior load. During posterior load, there were two records, one to the dentate and one to the edentulous area. For each record, one model was used to avoid the development of internal stress in the models, which may influence the quality of the actual strain. Due to the genuine limitation of DIC regarding the maximum strain thickness that the CCD camera can capture, which is 3–4 mm, the details of strains around the root of the abutments and the deeper area of the defect were not applicable. So, the analysis included only the strain around the available area, including the alveolar process of the abutments on the dentate side and the superior border of the defect.

The data were qualified and quantified using data processing and strain gauge options in “Lavision software.” For qualitative strain evaluation, the strain was analyzed using color mapping, where positive values (ranging from yellow to red) indicated tensile strains, while negative values (ranging from green to blue) represented compressive strains [19,27,28]. The quantitative data were imported to SPSS software (IBM SPSS Statistics, v22; IBM Corp; Armonk, New York, USA), assessed for normality using the Shapiro-Wilk test, and then tested using the Kruskal-Wallis and Mann-Whitney tests. A P-value less than.05 was used to reject the null hypothesis.

### Evaluation of strain using FEA

To evaluate support and strain by FEA, a laboratory scanner (3D scanner; NextEngine) was used to scan one of the models to import the data into Meshmixer software for processing. On the model, the assigned designs were sketched, isolated, and emitted to adjust the thicknesses of the bases to be 2 mm for the acrylic resin and 0.7 mm for the metal [38]. The Mimics program was used to segment the teeth from the skull, which were then imported into the Meshmixer program and repositioned to their proper position inside the model. Thereafter, the mucosa, periodontal ligament, cortical and cancellous bones were all imitated to be 2 mm of mucosa, 0.2 mm for periodontal ligament, 1 mm of cortical bone, and the remaining portion was regarded as cancellous bone [39,40]. Regarding the prostheses, one of the finalized obturators was scanned and imported to Meshmixer to be adapted to the defective part. All files were then imported into the 3-Matic software (3-Matic Innovation Suite; Materialize; New York, USA) for justifying the surface irregularities, then imported to Workbench software (ANSYS 2023R2; ANSYS Inc; Canonsburg, USA) for adding the materials properties [Table 1], meshing using elements of 4-node 3-D tetrahedral with a result of nodes of numbers 2,133,577.0, 1,935,433.0, 1,697,520.0, and 1,784,732.0, and elements of 1,365,410.0, 1,00,905.0, 839327.0, and 954,282.0 for ARO, LDO, TDO, and FTDO, respectively. The central incisor and molar areas were chosen to receive two types of loads, including one vertical and one oblique load, with 100 N for each [33,41]. The vertical loads were directed to the edge of the central incisor and occlusal table of the molars. The oblique loads were angled at 30 degrees toward the facial side and directed toward the palate.

**Table 1:** The properties of the materials used in the current study.

Materials	Young’s modulus (MPa)	Poisson’s ratio
Teeth (simulated by enamel) [42]	80,000	0.30
Periodontal ligament [42]	175	0.45
Mucosa [43]	3.45	0.40
Cancellous bone [43]	1370	0.30
Cortical bone [43]	13,700	0.30
Co–Cr alloy [44]	220,000	0.33
Acrylic resin [43]	2200	0.35

Using the ANSYS Workbench program, descriptive statistics of von Mises strain value and the associated displacement (lack of support) were evaluated to accurately forecast the strain distribution of prosthetic parts and supporting structures [45]. In quantitative terms, designs with elevated von Mises strain values correlated with an increased likelihood of bone resorption, while those exhibiting more significant displacement indicated reduced structural support, and conversely, lower displacement or strain corresponded to improved stability and lower resorption risk [46,47]. The location and intensity of strain and displacement were qualitatively identified through color mapping [33].

## Results

**Fig 5:**
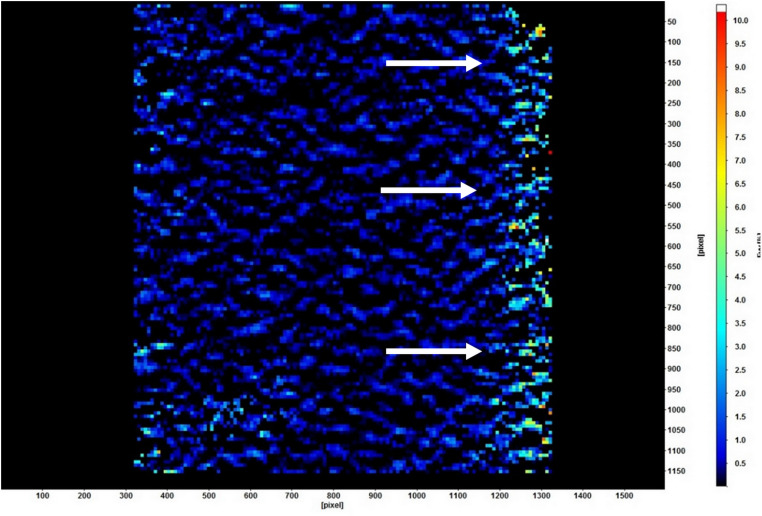
Strain distribution in the anterior area of linear design obturator under 150 N vertical loading using digital image correlation. The strain was concentrated along the alveolus of teeth next to the edentulous area (the white arrows).

**Fig 6:**
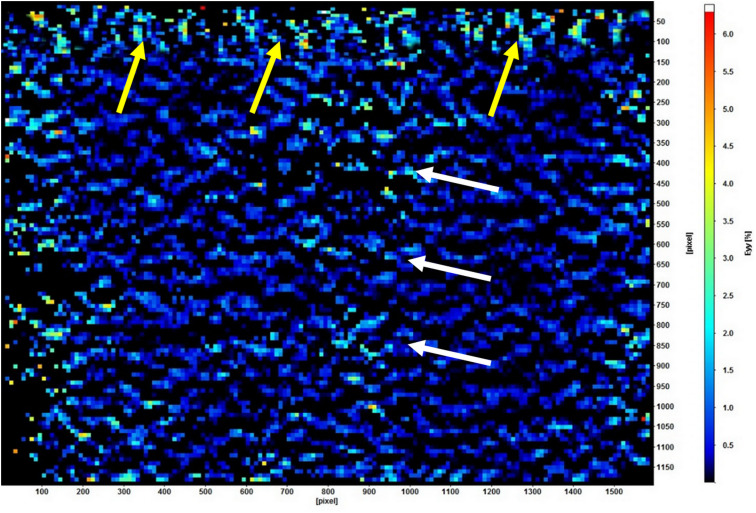
Strain distribution in the dentate side of linear design obturators under 150 N vertical loading using digital image correlation. The strain is distributed over the alveolar surface of posterior teeth (yellow arrows) and extends between the first and second premolars (white arrows).

Table 2 shows the strain distribution in the supporting structures using DIC. The acrylic resin-based showed the lowest strain (2.18 × 10^-3^), followed significantly by the TDO obturators (26.19 × 10^-3^; *P* < .001). The LDO showed the highest strain concentration (72.94 × 10^-3^; *P* < .001), followed insignificantly by the FTDO (69.81 × 10^-3^; *P* = .436). The strain was mainly concentrated at the alveolus of the incisors and the adjacent supporting bone (Fig 5). Regarding posterior vertical load, the alveolar bone of the dentate side of the ARO demonstrated the lowest strain (14.14 × 10^-3^; *P* = .007), followed insignificantly by the TDO and FTDO. In contrast, the LDO showed the highest strain (32.02 × 10^-3^; *P* < .001). The strain was concentrated on the premolars extending to their roots (Fig 6). Regarding the supportive bone of the defective side, the strain significantly increased on the edentulous side compared to the anterior and dentate sides. The TDO demonstrated the lowest strain (13.02 × 10^-3^ *P* < .001), followed significantly by the FTDO (21.18 × 10^-3^; *P* < .001). Entirely, the highest strain was caused by the acrylic resin-based obturators (1261.3 × 10^-3^; *P* < .001), followed significantly by the LDO (121.96 × 10^-3^; *P* < .001). The strain was concentrated at the lateral and anterior walls of the defect.

**Table 2:** The strain distribution in the supporting structure (anterior teeth, dentate, and defect side) of various obturators under 150 N loading, using digital image correlation.

Area of load application	Obturators with assorted designs Kruskal-Wallis and Mann-Whitney tests				*P* value
	ARO	LDO	TDO	FTDO	
	Mean±SD ×10^-3^	Mean±SD ×10^-3^	Mean±SD ×10^-3^	Mean±SD ×10^-3^	
Anterior area	2.18 (1.70)^c^	72.94 (66.93)^a^	26.19 (19.54)^b^	69.81 (30.72)^a^	≤.001^*^
Dentate side	14.14 (8.04)^b^	32.02 (27.10)^a^	18.56 (15.30)^b^	18.24 (14.10)^b^	≤.001^*^
Defective side	1261.30 (724.76)^a^	121.96 (98.08)^b^	13.72 (11.26)^d^	20.62 (17.70)^c^	≤.001^*^
The entire prosthesis	259.09 (585.44)^b^	69.57 (72.38)^a^	19.94 (19.83)^c^	34.90 (29.86)^c^	≤.001^*^

ARO: acrylic-resin-based obturators, LDO: linear, TDO: tripodal, FTDO: fully tripodal, SD: the standard deviation “a-d” shows the statistical differences while [a] is the highest and [d] is the lowest. Similar letters show no significant differences between the corresponding variables.

**Fig 7:**
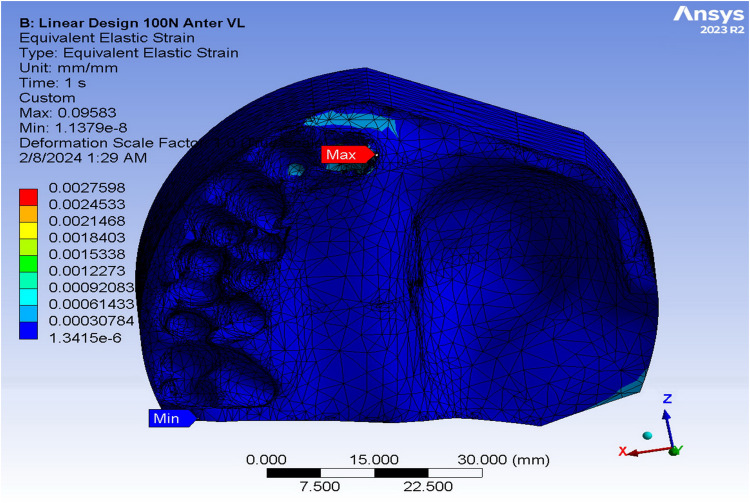
Strain distribution in the supporting bone under linear design obturators upon 100 N anterior vertical loading using finite element analysis.

**Fig 8:**
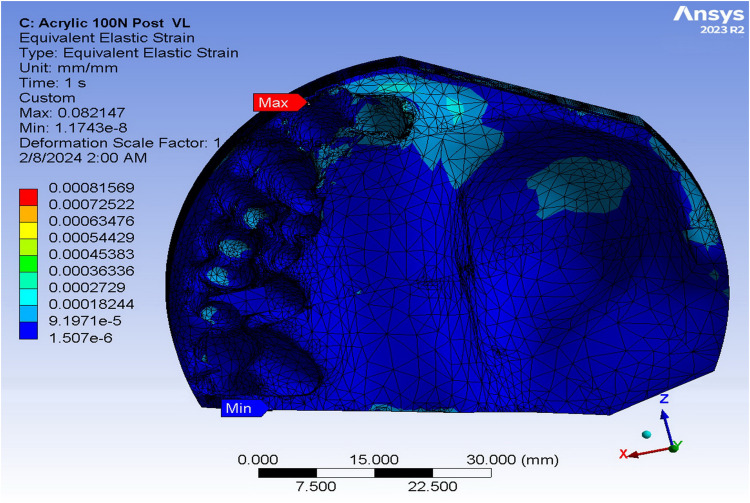
Strain distribution in the supporting bone under acrylic resin-based obturators under 100 N posterior vertical loading using finite element analysis.

Table 3 shows the quantitative values of strain in various obturators subjected to anterior loads of 100N using FEA. The supporting bone of the ARO displayed the highest strain values in the vertical load (3.06 × 10^-3^), while the TDO revealed the highest strain in the oblique load (3.61 × 10^-3^). The FTDO substantiated the lowest strain values in response to the vertical loads (1.77 × 10^-3^), while the ARO validated the lowest strain upon the oblique loads. The strain was distributed mainly in the buccal and apical alveolus of the central and lateral incisors (Fig 7). Adams clasps of ARO showed the highest strain values in the vertical load, while the framework of the TDO showed the highest strain in the oblique load (0.53 × 10^-3^). The FTDO expressed the lowest strain values in different applied loads (0.32 and 0.23 × 10^-3^ for vertical and oblique loads, respectively). Regarding the von Mises strain values on the different obturators and their supporting structures upon posterior loadings, the jaw and alveolar bone of the TDO expressed the highest strain value under vertical and oblique loads, while the ARO corroborated the least. The strain was mainly concentrated in the mesial side of the central incisor and base of the defect (Fig 8). After examining the framework model, the TDO framework manifested the highest strain, followed by FTDO, while the ARO showed the lowest.

**Table 3:** Strain distribution of supporting structures and prosthetic components of various obturators under anterior and posterior loading using finite element analysis.

Supporting structures	Anterior load of 100 N							
	Vertical load (×10^–3^)				Oblique load (×10^–3^)			
	ARO	LDO	TDO	FTDO	ARO	LDO	TDO	FTDO
Bone	3.06	2.76	1.82	1.77	1.69	1.78	3.61	1.84
Framework	0.72	0.70	0.53	0.32	0.24	0.48	0.53	0.23
Prosthetic portion	27.51	95.83	49.82	73.23	5.72	32.17	24.36	13.49
Supporting structures	Posterior load of 100 N							
	Vertical load (×10^–3^)				Oblique load (×10^–3^)			
	ARO	LDO	TDO	FTDO	ARO	LDO	TDO	FTDO
Bone	0.82	1.12	2.62	1.54	0.51	1.52	3.27	1.72
Framework	0.13	0.27	1.36	0.96	0.19	0.31	1.44	0.55
Prosthetic portion	15.10	43.33	33.53	25.37	8.88	29.45	19.06	42.07

FEA: finite element analysis, ARO: acrylic-resin-based obturators, LDO: linear, TDO: tripodal, FTDO: fully tripodal, SD: the standard deviation

**Fig 9:**
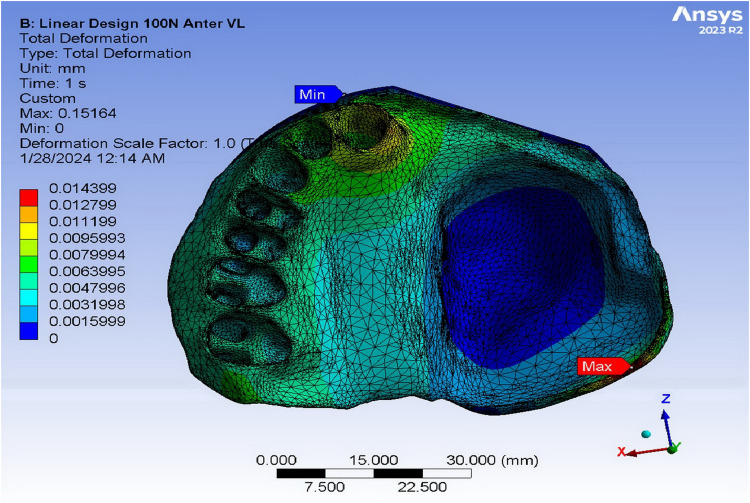
The total deformation in the supporting bone under linear design obturator upon 100 N anterior vertical loading using Finite Element Analysis.

Table 4 demonstrates values of deformation or the tissue ward movement of the various obturators and supporting structures upon loads of 100 N using FEA. The LDO demonstrated the highest deformation upon the anterior vertical and oblique loads, while the ARO expressed the least. The entire supporting bone showed the highest displacement in the LDO, followed by ARO. The displacement was directed toward the anterior palatal bone and posterior lateral wall of the defect (Fig 9). Under posterior loads, the abutments and their periodontal ligament of LDO design showed the lowest displacement (highest support) in both vertical and lateral loads, followed by the TDO (under vertical load) and the FTDO (under oblique load).

**Table 4:** The deformation in the supporting structure and the tissue-ward movement in the various maxillofacial prostheses upon 100 N anterior and posterior loading using FEA.

Supporting structures	Anterior load of 100 N							
	Vertical load (×10^–3^ mm)				Oblique load (×10^–3^ mm)			
	ARO	LDO	TDO	FTDO	ARO	LDO	TDO	FTDO
Dental	19.12	11.55	14.65	15.57	19.7	10.37	16.10	14.50
Periodontal ligament	14.16	10.85	11.82	12.37	12.67	9.57	10.81	10.60
Mucosa	23.15	49.13	18.98	17.95	19.24	31.76	13.39	12.11
Bone	13.82	14.40	12.25	12.62	12.05	13.18	10.78	10.51
Framework	8.91	22.30	16.31	16.26	7.85	17.62	13.33	12.33
Prosthetic portion	66.50	151.64	79.69	86.06	50.57	82.58	76.67	33.75
Supporting structures	Posterior load of 100 N							
	Vertical load (×10^–3^ mm)				Oblique load (×10^–3^ mm)			
	ARO	LDO	TDO	FTDO	ARO	LDO	TDO	FTDO
Dental	11.98	6.89	7.95	8.07	16.89	12.32	15.55	15.03
Periodontal lgiament	7.17	5.61	6.26	6.39	11.24	9.58	11.01	10.88
Mucosa	67.28	63.66	50.41	50.33	77.05	72.18	60.60	59.80
Bone	6.391	18.20	15.62	16.43	10.56	25.83	18.37	23.17
Framework	9.08	23.35	18.99	18.91	17.22	27.60	20.96	25.48
Prosthetic portion	84.99	86.01	68.75	65.74	104.51	116.42	98.04	112.63

FEA: finite element analysis, ARO: acrylic-resin-based obturators, LDO: linear, TDO: tripodal, FTDO: fully tripodal, SD: the standard deviation

## Discussion

Using DIC and FEA, the strain and displacement in removable obturators with various designs were evaluated. The study demonstrated significant differences in the strain and displacement in both methods, which led to the rejection of the assigned null hypothesis. In DIC, one vertical load was applied anteriorly and posteriorly. Three models were used to assess the strain from three views: one from the anterior view during anterior loading, one from the dentate side, and one from the defect side during posterior loading. Using FEA, two loads were applied to the anterior and posterior areas, including one vertical and one oblique. The oblique loads were added in the finite element models to express the forces developed during the function of anterior and posterior teeth.

In DIC, the entire ARO produced the highest strain distribution in the supporting structure (alveolus), followed by the LDO. The TDO had the lowest strain, followed by the FTDO. These results were nearly identical to FEA, where the ARO demonstrated the highest strain in the anterior vertical loads. That could be explained by the characteristics of the acrylic resin properties that demonstrate more flexibility than the metal, which leads to bending or displacement of the prosthesis toward the supporting bone. The configuration of the occlusal rests, the broad tissue coverage of the major connector, and the mechanical properties of the cobalt-chromium material could explain the lowest strain in the TDO and the FTDO, as mentioned in two studies that examined the influences of the zygomatic implant on obturators restoring unilateral maxillary defects [48,49]. The same cause could explain why TDO demonstrated the highest strain within the bone upon anterior oblique loads, especially the teeth next to the defect. The less tissue coverage (in the LDO and the FTDO) or the acrylic resin properties (in ARO) could explain why the three designs produce less strain during anterior oblique load than the TDO.

Under the posterior loading, DIC demonstrated that the alveolar bone of the dentate side of the LDO showed the highest strain distribution compared to the other designs. That showed inconsistency with the results of FEA, which showed that the TDO showed the highest, but ARO had the lowest strain in both approaches. However, the variation in the strain values between the metal-based designs was minor in FEA data. The result presented by DIC was explainable as the support was received from fewer abutments, which may increase the strain in their alveolar bone. The lowest strain demonstrated by the ARO was also explainable because no actual supportive components were placed on the abutments besides the high flexibility of the acrylic resin materials. Hence, the strain in the alveolar bone in the ARO was minor. On the same base, the ARO demonstrated the highest strain distribution on the supporting bone of the defect area, followed by LDO, using the DIC. That was explainable by the high flexibility of acrylic resin materials and the lack of occlusal rests.

Regarding support, the LDO demonstrated the highest displacement in anterior and posterior forces compared to the other metal prostheses. That can be explained by the configuration of the occlusal rests of LDO compared to those used for TDO and FTDO. Also, the FTDO showed a minor increase in displacement compared to the TDO obturators. That may be due to the less coverage of the FTDO compared to the TDO obturators, which may cause a minor increase in flexibility.

Biological variations in the cases of unilateral defects, the condition of remaining abutments, the number and their periodontal conditions, the defect configurations, the patient classification, oral hygiene practices, and post-treatment care may limit the present study. However, additional clinical research to compare and assess the clinical dependability of assorted obturators may overcome these limitations.

## Conclusion

Within the limitations of the current study, the following can be stated

1. The acrylic resin-based obturator produces the highest total strain compared to other obturators, while the tripodal design produces the lowest.
2. The linear design obturators demonstrate the lowest support and the highest von Mises strain value on the alveolar process of the anterior abutment compared to the tripodal and fully tripodal design obturators.
3. Although the deformation of the fully tripodal design obturators was high compared to the tripodal one, the overall strain of both designs was comparable.
4. Acrylic resin-based obturators still provide adequate treatment options from a biomechanical point of view.
5. DIC and FEA were nearly comparable regarding the support and strain distribution of the various obturators.

## Supporting information

S1 Data The basic raw data. (XLSX)

## Abbreviations

DIC: Digital Image correlations
FEA: Finite Element analysis
ARO: Acrylic resin-based obturators
LDO: Linear design obturator
TDO: Tripodal design obturator
FTDO: Fully tripodal design obturator.
